# Mapping fibrosis in colorectal liver metastases (CRLM) with gadobenate dimeglumine-enhanced MRI: prognostic implications and imaging biomarkers

**DOI:** 10.1007/s00384-026-05108-8

**Published:** 2026-02-19

**Authors:** Irmina Morawska, Katarzyna Pasicz, Andrzej Cieszanowski

**Affiliations:** 1 1st Department of Radiology, The Maria Sklodowska-Curie National Research Institute of Oncology, 5 Wilhelm Conrad Roentgen St, 02-781 Warsaw, Poland; 2 Department of Medical Physics, The Maria Sklodowska-Curie National Research Institute of Oncology, 5 Wilhelm Conrad Roentgen St, 02-781 Warsaw, Poland; 3 2nd Department of Clinical Radiology, Faculty of Medicine, Medical University of Warsaw, 1 A Stefan Banach St, 02-097 Warsaw, Poland; 4 Department of Biomedicine, Neuroscience and Advanced Diagnostics (BiND), University of Palermo, Via del Vespro 129, 90127 Palermo, Italy

**Keywords:** Tumor-to-liver enhancement index, Signal intensity change percentage, Fibrosis, Colorectal liver metastases, Chemotherapy, Gadobenate dimeglumine (Gd-BOPTA)-enhanced MRI

## Abstract

**Purpose:**

Development of fibrosis in treated colorectal liver metastases (CRLM) could be supposedly used for the estimation of both treatment response and prognosis. This study aimed to investigate the association between post-chemotherapy, fibrosis-related progressive gadolinium enhancement of CRLM on MRI and overall survival.

**Material and methods:**

A retrospective study of 97 CRLM patients (68 M, mean age 62.3 ± 10.71 years) who underwent between 2017 and 2022 preoperative gadobenate dimeglumine (Gd-BOPTA) – enhanced MRI after chemotherapy. Tumor and liver enhancement were quantified using Signal Intensity Change Percentages (SICP) across 5-min and 60-min delay phases, along with the Tumor-to-Liver Enhancement Index (TLEI) to estimate fibrosis within CRLM. A subset of 18 patients was evaluated for radiologic-pathologic correlation. Cox regression, Kaplan–Meier analysis, and multivariate models were used to assess overall survival (OS).

**Results:**

High SICP (≥ 90.3%) in the 60-min delayed phase was associated with significantly lower OS (median: 37 vs. 66 months; *p* = 0.023). TLEI was significantly elevated in non-survivors (1.25 vs. 1.10; *p* = 0.007). Histopathologic correlation, available in 18 patients, confirmed fibrosis in lesions with elevated SICP, though limited sample size precluded statistical validation. In multivariate analysis, both high TLEI and elevated SICP were independent predictors of reduced OS (HR 1.38 [1.05–1.82], *p* = 0.023; HR 1.01 [1.00–1.01], *p* = 0.043, respectively). Notably, aflibercept- and FOLFOX-4-treated patients showed higher fibrosis-associated enhancement.

**Conclusion:**

Gd-BOPTA-enhanced MRI, specifically SICP and TLEI in the delayed phase, may serve as non-invasive imaging biomarkers of fibrosis in CRLM. Contrary to prior assumptions, increased fibrosis was associated with worse prognosis, suggesting fibrosis-mediated tumor microenvironment alterations. Prospective studies with robust radiologic-pathologic validation are needed to clarify the mechanistic and prognostic implications.

**Supplementary Information:**

The online version contains supplementary material available at 10.1007/s00384-026-05108-8.

## Introduction

Colorectal liver metastases (CRLM) are the most prevalent and critical manifestation of metastatic colorectal cancer (CRC) [1, 2]. Recent advancements in systemic therapies, particularly combining chemotherapy with targeted agents, have led to improved survival rates, which can vary from 30 to 50 months based on factors such as tumor biology and treatment approaches. Nevertheless, there is still significant variability in patient outcomes, even among those receiving similar treatment regimens [2, 3].

Fibrosis has traditionally been regarded as a positive sign of treatment-induced regression in CRLM, largely based on histopathological findings [4]. The development of fibrous tissue within metastases is typically interpreted as an indication of effective chemotherapy-induced tumor necrosis and subsequent scarring [4, 5]. However, the evaluation of fibrosis has primarily relied on histopathology, with its imaging correlates remaining inadequately defined.

Magnetic resonance imaging (MRI) is a commonly utilized imaging technique for assessing liver metastases in colorectal cancer patients receiving chemotherapy [6]. However, its efficacy in evaluating fibrosis within metastatic lesions is limited. This limitation is mainly due to the overlapping signal characteristics between fibrotic and viable tumoral tissues, as well as the impact of post-therapy changes such as necrosis and altered vascularity [6]. Moreover, conventional MRI sequences may not possess sufficient sensitivity to distinguish these tissue types [7]. One potential imaging marker of fibrosis is progressive enhancement – a gradual increase in lesion signal intensity on delayed post-contrast sequences – which reflects the accumulation of contrast agents within the extracellular matrix [4]. This pattern has been associated with fibrotic tissue in both primary and metastatic tumors and may offer a non-invasive surrogate for histopathological fibrosis [8].

The development of fibrosis in treated CRLM could potentially serve as a marker for assessing both treatment response and prognosis [6]. Our study aims to fill this gap by providing the first quantitative evaluation of chemotherapy-induced fibrosis in CRLM using gadobenate dimeglumine (Gd-BOPTA) – enhanced MRI. To achieve an objective and reproducible method for quantifying fibrosis, we adapted and modified imaging parameters that have been previously validated in renal oncology. Aydoğan et al. showcased the effectiveness of the Signal Intensity Change Percentage (SICP) and Tumor-to-Cortex Enhancement Index (TCEI) in assessing diagnostic performance in clear cell renal cell carcinoma (ccRCC) and non-clear cell carcinoma (non-ccRCC) through multiparametric MRI [9]. These tumor subtypes exhibit significant differences in their fibrotic composition: ccRCC is notably vascularized with a clear histological appearance and typically shows minimal fibrosis, except in advanced cases featuring necrosis or hemorrhage, where secondary fibrotic changes can develop [10–13]. Conversely, non-ccRCC subtypes, including chromophobe RCC (chRCC) and papillary RCC (pRCC), often display more substantial fibrotic elements, characterized by thicker fibrous septa and stromal connective tissue [10–13]. The study established that delayed gadolinium enhancement reflects extracellular matrix expansion, making these parameters suitable for evaluating fibrotic changes in malignancies [4]. In our study we adapted these metrics in the context of metastatic CRC.

Moreover, based on previous publications we presumed that the degree of fibrosis may be also assessed during hepatobiliary phase after injection of gadobenate dimeglumine [4, 8, 14].

Therefore, by applying this methodology to CRLM, we aimed to develop a quantitative, MRI-based tool for assessing fibrosis that is both scientifically robust and clinically applicable. We introduced and validated two new imaging biomarkers – SICP and Tumor-to-Liver Enhancement Index (TLEI) during 5-min and 60-min delayed phases – to evaluate the relationship between MRI-assessed fibrosis and histopathological fibrosis, as well as to investigate the prognostic significance of fibrosis quantification in predicting overall survival in CRLM patients.

## Material and methods

### Study population

As this was a retrospective study, informed consent was not obtained from the participants. Approval for the study was granted by the Maria Sklodowska-Curie National Research Institute of Oncology Ethics Committee in Warsaw, Poland. We followed The Strengthening the Reporting of Observational Studies in Epidemiology (STROBE) guidelines to enhance the study's quality [15]. Our research group consisted of 97 consecutive patients with CRLM at a single medical institution (68 males, mean age: 62.3 ± 10.71 years) who underwent Gd-BOPTA– enhanced MRI following chemotherapy. The study cohort was categorized into two primary comparison groups based on fibrosis levels classified into low SICP (< 90.3%) and high SICP (≥ 90.3%) categories according to the proportion of fibrotic tissue identified in the resected tumor specimen after neoadjuvant chemotherapy. Based on prior MRI biomarker validation in fibrotic renal malignancies, we adopted a cutoff of SICP ≥ 90.3% in the hepatobiliary phase to define high fibrotic burden [9]. This threshold was hypothesized to reflect extracellular matrix expansion and stromal remodeling, phenomena known to occur in chemoresistent metastatic CRC. Data were collected from patients examined in the 1 st Department of Radiology at the Maria Sklodowska-Curie National Research Institute of Oncology in Warsaw, Poland, from January 2017 to December 2022. The censoring date for survival analysis was December 31, 2022. The decision to initiate neoadjuvant chemotherapy for CRLM was made on a case-by-case basis, influenced by factors such as tumor resectability, patient performance status, and the extent of liver involvement and was discussed by the multidisciplinary team. Patient records were sourced from electronic health databases.

**Inclusion criteria** were as follows: patients with histopathological confirmation of CRC and CRLM; undergoing MRI scans post-preoperative chemotherapy and obtaining images of adequate quality with minimal imaging and motion artifacts in the regions of interest (ROI).

**Exclusion criteria** were established following the presence of multiple cancers simultaneously; liver metastases from cancers of unknown primary (CUP); patients with confirmed CRC but without liver metastases; examinations conducted on different scanners (1.5 T and 3 T); patients who had not received preoperative chemotherapy; death due to surgical or non-tumor-related complications; patients with CRLM too small for evaluating intratumoral fat on MR images; contraindications to MR examination and intravenous administration of paramagnetic contrast agents such as: implanted pacemaker/cardioverter-defibrillator, central nervous system vascular clips, auditory implant, insulin pumps, metallic foreign bodies in the eyeball, claustrophobia, significant renal insufficiency (eGFR < 30 ml/min) and finally transfer of the patient to another oncology center.

### Imaging protocol

MRI examinations for 71 patients were performed on a 3.0 T scanner (Signa Architect, GE or Skyra, Siemens), while 26 patients were imaged on a 1.5 T scanner (Aera, Siemens). Variability in SICP group definitions between scanners or timepoints was minimized by applying fixed thresholds. However, subtle inconsistencies in ROI placement or signal interpretation may persist; these were mitigated by consensus review by two radiologists. The time interval between the last cycle of chemotherapy and MRI examination was recorded for all patients. To minimize variability, scans were performed within a standardized window of 4–6 weeks after treatment completion, corresponding to the typical recovery period from chemotherapy-induced effects while ensuring residual therapeutic changes remained detectable. The progressive enhancement MRI protocol included T1-weighted 3D gradient-echo sequences acquired before and after intravenous administration of the hepatobiliary gadolinium-based contrast agent gadobenate dimeglumine (Gd-BOPTA, MultiHance®, Bracco Imaging, Milan, Italy). Imaging parameters were optimized to ensure the best contrast-to-noise ratio and spatial resolution, with acquisition settings summarized in Tables 1 and 2. To address inter-scanner variability, we implemented the following harmonization steps: SICP and TLEI were computed as relative signal intensity ratios, normalized to pre-contrast values, thus minimizing dependence on absolute signal levels; all data were processed on a single post-processing workstation using uniform settings for ROI intensity extraction and regular phantom calibration was performed at 3-month intervals across scanners to ensure signal stability.

**Table 1:** MRI acquisition parameters across scanners and harmonization procedures

Parameters	*Aera 1.5 T* *(Siemens)*	*Skyra 3 T* *(Siemens)*	*Signa Architect 3 T* *(GE Healthcare)*
Field Strength (T)	1.5	3.0	3.0
Scanner Model	MAGNETOM Aera	MAGNETOM Skyra	Signa Architect
Manufacturer	Siemens	Siemens	GE Healthcare
Sequence (Pre/Post Contrast)	T1-weighted 3D GRE (VIBE)	T1-weighted 3D GRE (VIBE)	T1-weighted 3D GRE (LAVA-XV)
Imaging Plane	Axial	Axial	Axial
Repetition Time—TR (ms)	3.5–5.0	3.0–4.5	4.0–5.5
Echo Time—TE (ms)	1.2–2.5	1.0–2.0	1.5–2.5
Flip Angle (°)	10–15	10–12	12–15
Image Matrix (%)	256 × 192 to 320 × 224	320 × 224 to 384 × 256	320 × 224 to 384 × 288
Slice Thickness (mm)	3–4	3–4	3–4
Inter-slice Gap (mm)	0–1	0–1	0–1
Field of View (FOV) (mm)	320–400	350–420	350–420
Bandwidth (Hz/pixel)	300–500	350–550	400–600
Parallel Imaging Technique	GRAPPA (acceleration 2)	GRAPPA (2)	ARC (2)
Fat Suppression Method	SPAIR	SPAIR	IDEAL/Dixon
Contrast Agent	Gd-BOPTA	Gd-BOPTA	Gd-BOPTA
Contrast Dose	0.1 mmol/kg	0.1 mmol/kg	0.1 mmol/kg
Injection Rate	1.5 mL/s	1.5 mL/s	1.5 mL/s
Saline Flush	30 mL	30 mL	30 mL
Delayed Phase Timing	5 min & 60 min	5 min & 60 min	5 min & 60 min
Vendor-specific Notes	Siemens VIBE sequence optimized for extracellular contrast	Same protocol architecture as Aera	GE LAVA-Flex with Dixon separation; slight baseline SI differences addressed by normalization
Harmonization and Standardization Procedures			
Standardization Parameter	Implementation Across All Scanners		
Signal Normalization	SICP and TLEI computed as percentage change relative to pre-contrast SI, reducing dependence on TR/TE and receive gain		
Cross-scanner Alignment	All images processed on the same workstation with identical window/level presets and ROI protocol		
Phantom Calibration	Quarterly phantom scans performed on all scanners to ensure temporal signal stability		
ROI Placement Standardization	ROIs placed in identical hepatic segments and identical delayed phases (5 and 60 min)		
Post-processing Normalization	Use of relative rather than absolute SI metrics to mitigate inter-vendor differences (Siemens vs GE)		
Fixed Threshold Definition	Fixed SICP cutoffs applied after normalization, preventing threshold drift between scanners

**Table 2:** Additional MRI sequences

Sequence	Parameters
T2-weighted (Axial & Coronal)	Sequence: TSE/FSE Repetition Time (TR): 3000–5000 ms Echo Time (TE): 80–120 ms Slice Thickness: 5 mm Image Matrix (%): 320 × 256
Fat-suppressed T2-weighted	Spectral fat saturation or SPAIR technique
DWI	b-values: 0, 100, 500, 1000 s/mm^2^ Repetition Time (TR): 4000–6000 ms Echo Time (TE): 60–90 ms Image Matrix (%): 128 × 128 or 192 × 192

An adjusted dose of 0.1 mmol/kg gadobenate dimeglumine was administered intravenously at a rate of 2 ml/s, followed by a 20 ml saline flush. Dynamic contrast-enhanced imaging was performed in two key phases to evaluate tumor enhancement kinetics and contrast retention: 5-min delay and hepatobiliary phase (60-min delay). Additional images included axial and coronal T2-weighted, fat-suppressed T2-weighted, and diffusion-weighted images (DWI) with multiple b values (100, 500, 1000 s/mm^2^). Image data were stored in the PACS (Picture Archiving & Communication System, Centricity, GE HealthCare).

### Image analysis

**Fig. 1:**
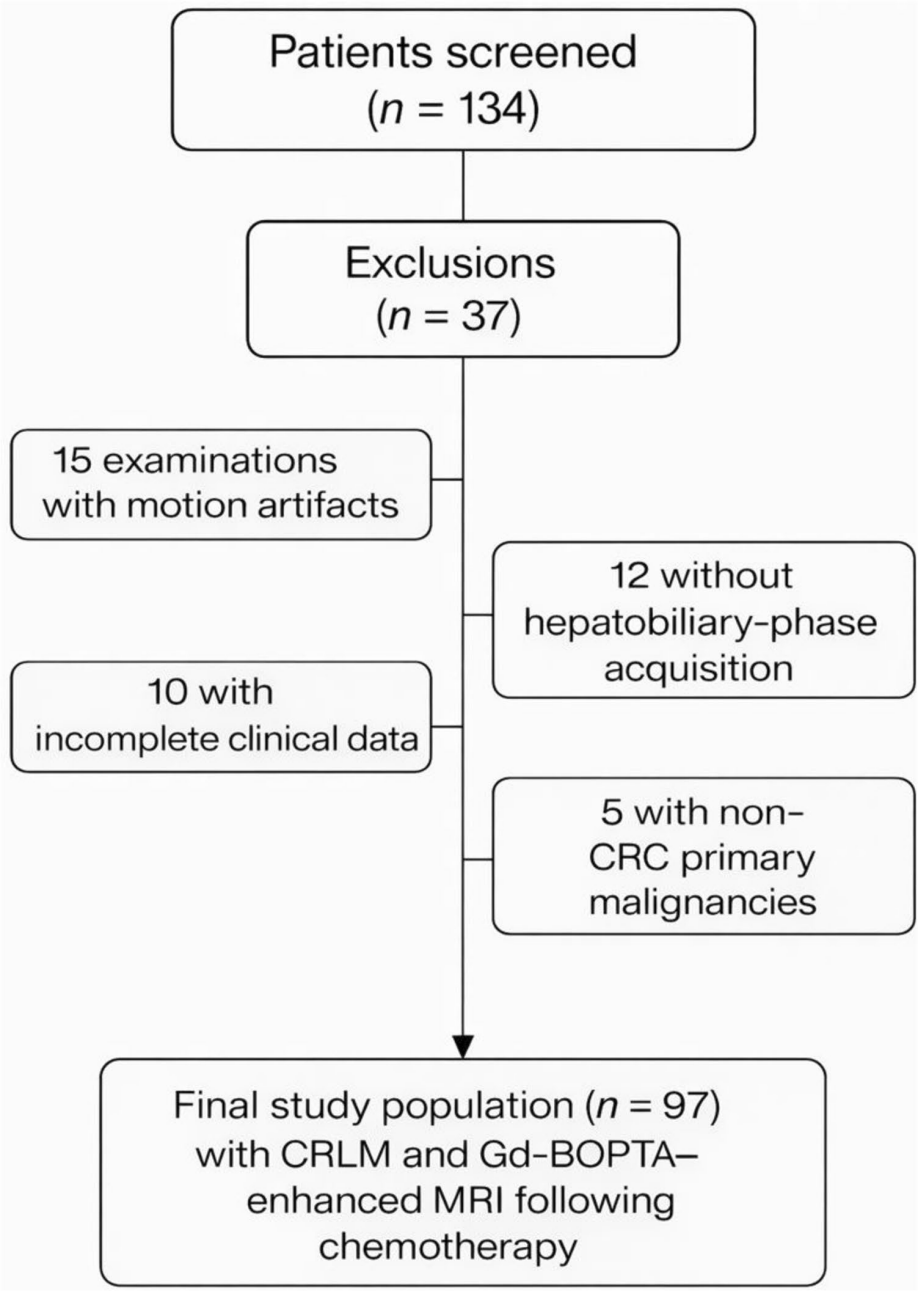
Flowchart of eligibility, exclusion criteria, and final study population

We evaluated 97 MRI scans individually [Fig. 1]. The assessment involved two experienced radiologists independently reviewing the images on PACS, with 20 and 5 years of experience in abdominal MRI, respectively. The radiologists were informed of CRC as the underlying disease but were blinded to the patients' personal data, results from other imaging modalities, and surgical or histopathological findings post-resection. Each liver segment was assessed to record the quantity and size of identified lesions, using liver maps and following the Coinaud classification [16].

Signal intensities of CRLM were determined by drawing an elliptic region of interest (ROI) as large as possible (minimum area of 12 mm^2^) to encompass the most enhanced region in CRLM for each patient. Circular ROIs were placed in both the tumor and normal liver parenchyma when evaluating dynamic contrast-enhanced sequences. Measurements were repeated on the normal liver parenchyma using the same ROI features. ROI measurements were consistently taken from the same side of the tumors and normal liver parenchyma across all phases. Based on contrast-enhanced images, solid regions without necrosis in the tumor were identified, and ROI measurements were recorded from three different points in these areas, averaging the results. This process was likewise applied to normal liver parenchyma.

In the dynamic contrast-enhanced MRI sequence, signal intensity change percentages (SICP) were calculated for both the tumor and normal liver parenchyma during 5-min delayed phase and hepatobiliary phase (60-min delayed) using the following formula [9]:

$$SICP={SI}_{post}-{SI}_{pre}/{SI}_{pre}\times 100$$

where SI_post_ represents the signal intensity (SI) during the contrast-enhanced phase, and SI_pre_ is the SI prior to contrast agent administration. The Tumor-to-Liver Enhancement Index (TLEI) was calculated by dividing the tumor SICP by the normal liver parenchyma SICP to assess the enhancement level of the tumor relative to the normal liver parenchyma. The threshold of SICP ≥ 90.3% for defining “high fibrosis” was selected based on prior studies in renal oncology, where similar delayed enhancement thresholds correlated with histopathologically confirmed fibrosis [9]. Given the analogous mechanism of extracellular contrast retention in fibrotic tissue, we applied this cutoff to CRLM. Lesions exhibiting significant artifacts (particularly in the left hepatic lobe or hepatic dome) were excluded from evaluation. The efficacy of chemotherapy was assessed by analyzing MRI results in accordance with RECIST 1.1 guidelines.

### Histopathological correlation

Gross tumor dimensions were measured as the maximum diameter following fixation in 10% buffered formalin. Haematoxylin and eosin (H&E)-stained slides were prepared from representative formalin-fixed paraffin-embedded (FFPE) tissue blocks. A gastrointestinal pathologist with over 10 years of experience qualitatively assessed the approximate proportions of fibrosis, necrosis, acellular mucin, and viable tumor cells on each representative slide. Two established frameworks were considered when defining the fibrosis assessment strategy for colorectal liver metastases (CRLM). The modified METAVIR scoring system, traditionally used to grade diffuse liver parenchymal fibrosis, provides a semi-quantitative evaluation of fibrotic deposition and architectural distortion ranging from F0 (no fibrosis) to F4 (cirrhosis). Although METAVIR does not directly apply to intra-tumoral fibrosis, its percentage-based approach to quantifying fibrotic expansion informed the structure of the adapted system used in this study. Similarly, the Rubbia-Brandt chemotherapy-associated liver injury (CALI) grading scheme evaluates treatment-related stromal alterations after oxaliplatin- or irinotecan-based therapy using a 0–3 scale (0 = none; 1 = mild; 2 = moderate; 3 = marked fibrosis) [17]. While originally designed to describe parenchymal injury rather than tumor fibrosis, this framework captures chemotherapy-induced stromal remodeling and likewise provided conceptual guidance. Together, these systems informed the development of our semi-quantitative, percentage-based grading method tailored specifically to CRLM, enabling consistent categorization of intra-tumoral fibrosis in the present cohort. Because neither METAVIR nor Rubbia-Brandt systems directly apply to intra-tumoral fibrosis in CRLM, we implemented an adapted semi-quantitative scale based on the percentage of fibrotic stroma within the tumor: Grade 0 (low fibrosis): < 30% fibrotic content, Grade 1 (moderate fibrosis): 30–60% fibrotic content, and Grade 2 (high fibrosis): > 60% fibrotic content. This approach was used in prior CRLM pathological response studies and enables direct correlation with imaging-derived extracellular contrast retention. All assessments were performed by an experienced gastrointestinal pathologist blinded to imaging, and reviewed for internal consistency by a senior consultant pathologist.

Radiologic-pathologic correlation was performed in a subset of patients by matching individual colorectal liver metastases (CRLM) identified on histopathology with the target CRLM on imaging, based on concordant tumor size and location. Patients were excluded from this analysis if corresponding pathology specimens were unavailable or if multiple metastases of similar size were present within the same liver segment, precluding accurate per-lesion matching. In total, radiologic-pathologic correlation was feasible in 18 (18.6%) out of 97 patients. All imaging and histopathological assessments were performed by evaluators blinded to clinical outcomes, apart from the known diagnosis of CRLM. Although formal inter-observer agreement could not be assessed, all fibrosis evaluations were independently reviewed and qualitatively verified by a senior consultant pathologist for internal consistency. Future studies should incorporate quantitative inter-reader reproducibility metrics, such as intraclass correlation coefficients (ICC), to validate the robustness of SICP and TLEI measurements.

### Statistical analysis

Predictors of fibrosis occurrence were identified through multivariate analyses. The presence of fibrosis was correlated with overall patient survival. Groups were compared based on clinical characteristics and outcomes. Treatment regimens were categorized as: PFF – panitumumab + FOLFOX-4/FOLFIRI and AFF – aflibercept + FOLFOX-4/FOLFIRI for subgroup analyses based on targeted therapy combinations. Patient survival was assessed from the date of hepatic MRI acquisition after final chemotherapy until death or last follow-up. Recurrence-free survival (RFS) was calculated from the end of chemotherapy to first recurrence or last follow-up.

To explore whether the SICP threshold used for survival analyses could be supported by liver-specific data, an internal cutoff optimization was performed. Receiver operating characteristic (ROC) curve analysis was conducted using OS and RFS as clinical endpoints. The optimal cutoff was determined using the Youden index. ROC analysis identified an optimal SICP threshold of 89.8% for OS and 90.1% for RFS. Given the minimal numerical difference and to maintain consistency across analyses, the predefined cutoff of SICP ≥ 90.3% was retained for dichotomization. Importantly, sensitivity analyses demonstrated stable hazard ratios across a narrow cutoff range (88–92%), indicating robustness of the association rather than dependence on a single arbitrary threshold. Continuous variables were expressed as mean ± standard deviation or median (1st – 3rd quartile), while categorical variables were represented by absolute values and percentages. The Shapiro–Wilk test was utilized to verify normality, followed by group comparisons using either Student's two-sample t-tests or Mann–Whitney U tests, depending on data distribution. The χ^2^ test was employed to assess differences in proportions among various groups. Patient survival was analyzed using the Kaplan–Meier method, with survival rate comparisons made through the Log-Rank Test. Univariate and multivariate analysis using the Cox Proportional Hazards Model was conducted to identify survival time predictors, including the impact of fibrosis. Results were expressed as hazard ratios (HR) with a 95% confidence interval (CI). Given the exploratory nature of some analyses, p-values were interpreted descriptively without correction for multiple comparisons (Bonferroni). While this approach increases the risk of Type I errors, it was deemed appropriate for hypothesis generation in this initial investigation of SICP/TLEI biomarkers. Future validation studies should employ stricter correction methods to confirm significance thresholds. Statistical significance was determined for tests with a p-value of 0.05 or less. All analyses were performed using Jamovi version 2.5.5 software [14, 18–25].

## Results

### Fibrosis after chemotherapy

Table 3 outlines the characteristics of the individuals included in this study (*n* = 97). On average, each patient had 3.2 ± 2.8 CRLMs (range: 1–25), and one representative lesion per patient was used for histopathologic comparison when feasible. The median time between chemotherapy completion and MRI acquisition was 5.2 weeks (IQR: 4.1–6.3 weeks). No significant correlation was found between this interval and SICP values (Spearman's ρ = 0.12, *p* = 0.24), suggesting that the observed enhancement patterns were stable during the study period rather than representing transient treatment effects.

**Table 3:** Demographics and clinical features of the patients

Factor	High SICP ***n* = 21** no. (%) or mean ± SD or median	Low SICP ***n* = 76** no. (%) or mean ± SD or median	*p* value
Age [years]	63.8 ± 10.2	60.7 ± 11	0.211
Sex (male), no. (%)	18 (85.71%)	50 (65.79%)	0.734
Site of primary tumor, no. (%)			
Right colon	1 (3.45%)	10 (13.16%)	0.196
Left colon	20 (96.55%)	52 (68.42%)	
Rectum	0 (0.00%)	14 (18.42%)	
Dimension of CRLM [mm]	36.9 ± 20.4	31.1 ± 22.7	0.164
Number of CRLM lesions			
Median	5	4	0.302
Range	1–25	1–20	
Fluorouracil-based chemotherapy regimen, no. (%)			
oxaliplatin	13 (61.91%)	36 (47.37%)	
irinotecan	6 (28.57%)	12 (15.79%)	
oxaliplatin and irinotecan	1 (4.76%)	15 (19.74%)	
neither oxaliplatin nor irinotecan	1 (4.76%)	5 (6.58%)	0.515
two or more regimens	1 (4.76%)	8 (10.52%)	
Bevacizumab, no. (%)			
Yes	5 (23.81%)	6 (7.89%)	0.152
No	16 (76.19%)	70 (92.11)	
Anti-EGFR agent (Cetuximab/Panitumumab), no. (%)			
Yes	6 (28.57%)	13 (17.11%)	0.803
No	15 (71.43%)	63 (82.89%)	
SICP [%]	82.3 ± 11.2	38.9 ± 17.4	< 0.001
TLEI	1.26 ± 0.11	1.08 ± 0.09	0.003

**Fig. 2:**
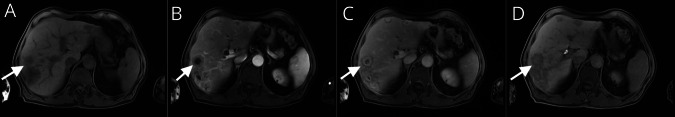
A 65-year-old man with CRLMs after FOLFOX-4 chemotherapy in gadobenate dimeglumine-enhanced MR imaging (A) (arrows). Progressive enhancement in the T1-weighted gradient echo magnetic resonance images starting with peripheral enhancement late arterial (B) and continuing in 5-min delay phase (C) with subsequent central enhancement in the hepatobiliary phase (D) corresponds with the high SICP (≥ 90.3%)

We identified a total of 307 CRLM in the study cohort. On MR images, the most substantial progressive enhancement with the highest SICP (exceeding 90.3%) in CRLM following contrast administration was qualitatively observed in 21 out of 97 (21.65%) patients post-chemotherapy [Fig. 2]. No statistically significant difference was found between the highest SICP and the number of CRLM (Spearman rank correlation, *p* = 0.802). Histopathologic correlation was feasible in 18 patients (18.6% of the cohort) and it confirmed the presence of a metastasis in all cases. Consequently, these radiologic-pathologic correlations should be interpreted as preliminary findings requiring validation in larger, prospectively matched cohorts. Among these cases, variable degrees of fibrosis were confirmed histologically: mild fibrosis was present in 8 patients, while significant fibrosis was observed in 10 cases. Baseline characteristics of these patients with available histopathology were compared to those without histopathological correlation [Table 4].

**Table 4:** Characteristics of patients with and without histopathological correlation

Factor	Histopatology Cohort ***n* = 18**	Full Cohort ***n* = 97**	*p* value
SICP [%]	82.3 [72.5–91.25]	68.9 [59.4–77.1]	0.011
Dimension (mm), mean ± SD	36.7 ± 19.2	32.4 ± 20.2	0.363
Age [years] mean ± SD	62.9 ± 10.1	60.2 ± 10.9	0.242
Sex, no. (%)			
Male	15 (83.3%)	68 (70.1%)	0.730
Female	3 (16.7%)	29 (29.9%)	
Chemotherapy regimen, no. (%)			
FOLFOX-4	13 (61.91%)	53 (54.6%)	
FOLFIRI	6 (28.57%)	12 (15.79%)	0.151
other	1 (4.76%)	15 (19.74%)	

### Overall survival

Chemotherapy regimens were heterogeneous: FOLFOX-4 (folinic acid (leucovorin or calcium folinate) combined with 5-fluorouracil and oxaliplatin) was administered to 53 (54.64%) of patients, while other regimens, including FOLFIRI (folinic acid (leucovorin or calcium folinate) combined with 5-fluorouracil and irinotecan) were administered to 24 (24.74%) patients, other regimens formed smaller subgroups 20 (20.61%). This distribution limited subsequent analyses of regimen-specific effects on fibrosis. Of the 12 (12.37%) patients treated with capecitabine, 4 (4.12%) also received oxaliplatin. Cetuximab and aflibercept were commonly administered alongside oxaliplatin. Patients treated with aflibercept and/or FOLFOX-4 exhibited significantly higher SICP (82.3% vs. 38.9%) and TLEI (1.26 vs. 1.08) compared to those receiving other chemotherapy regimens (*p* = 0.01).

Statistical differences were observed in SICP values and survival time. Only the SICP ≥ 90.3% at the 60-min delayed phase was statistically significant (*p* = 0.02). Both SICP (93.4% vs. 81.2%, *p* = 0.012) and TLEI (1.25 vs. 1.10, *p* = 0.007) varied between survivors (41.24%) and non-survivors (58.76%). Elevated SICP was associated with shorter survival [37 vs. 66 months, 95% CI]. Although histopathological correlation was available only in a limited number of cases, our findings suggest that SICP values ≥ 90.3% in the 60-min hepatobiliary phase may be associated with increased fibrotic content within CRLM.

**Fig. 3:**
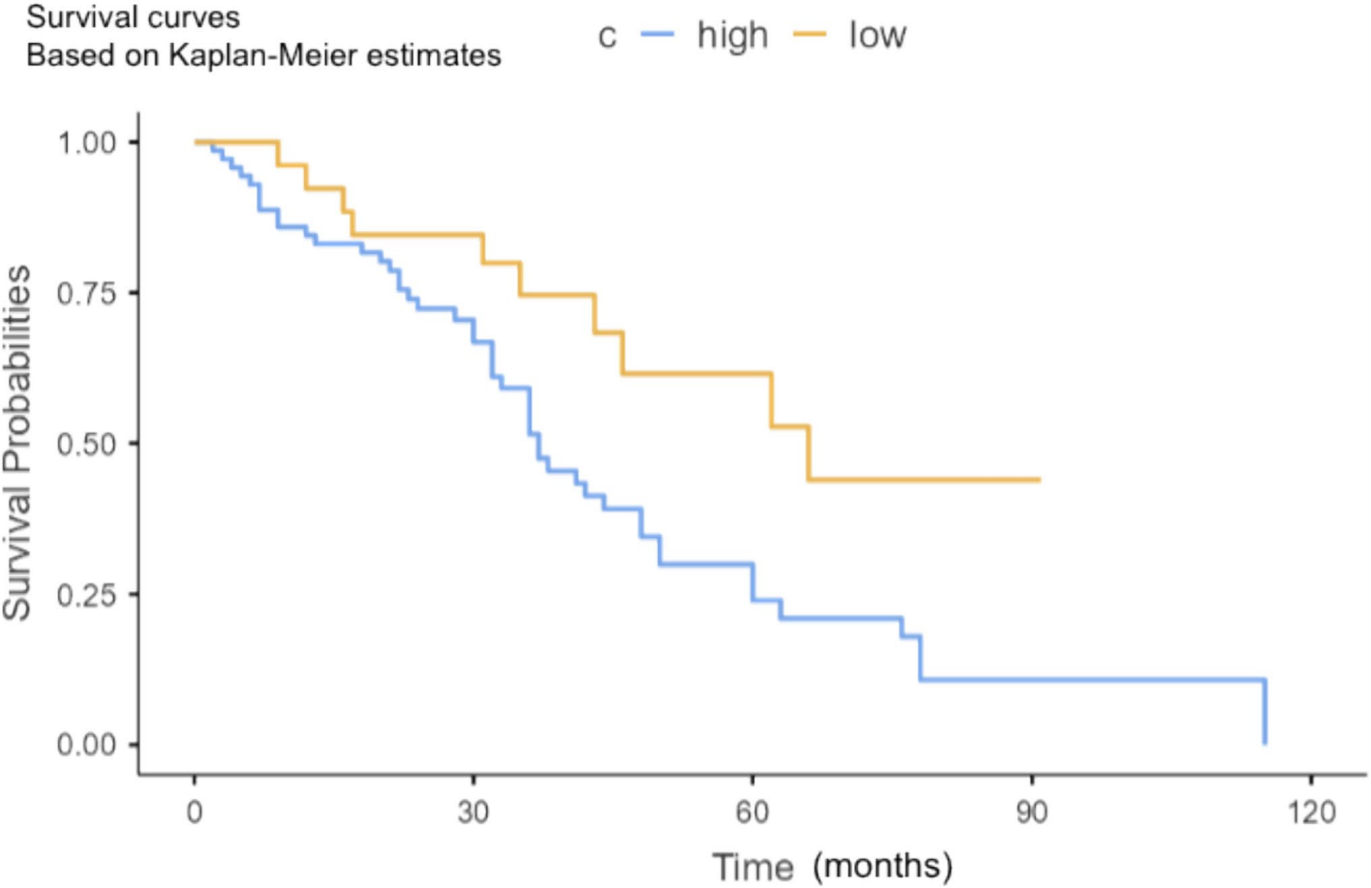
Multivariate survival analysis of patients with CRLM presenting high SICP values

The median follow-up duration for the entire cohort was 32 months. The Kaplan–Meier curve analysis revealed poorer overall survival in patients with higher SICP (OR 0.45 (0.23–0.90, *p* = 0.023) [Fig. 3]. In patients exhibiting elevated SICP values in CRLM, overall survival was shorter [37 months (24—60, 95% CI)] compared to those with lower SICP [66 months (36—NA, 95% CI)]. We observed differential effects by chemotherapy regimen type between patients receiving anti-EGFR-based combinations compared to anti-angiogenic regimens summarized in Table 5. Kaplan–Meier analysis showed significantly shorter RFS in patients with SICP ≥ 90.3% (median 22 vs. 44 months, log-rank *p* = 0.017). In multivariate Cox regression analysis for recurrence-free survival (RFS), including SICP category, chemotherapy regimen, number of lesions, maximum lesion diameter, histopathologic fibrosis grade, and age, SICP ≥ 90.3% remained an independent predictor of shorter RFS (HR 1.54, 95% CI 1.05–2.31, *p* = 0.029). Lesion number was also independently associated with shorter RFS (HR 1.12 per lesion, 95% CI 1.03–1.22, *p* = 0.008), whereas maximum lesion diameter, fibrosis grade, and chemotherapy regimen did not reach statistical significance in the multivariate model (all *p* > 0.05). Multiple factors such as different chemotherapy regimens (FOLFOX-4, FOLFIRI in conjunction with aflibercept or panitumumab), age, SICP, and TLEI in the delayed phase affecting survival. The multivariable-adjusted hazard ratios (HR) for age and various chemotherapy regimens are close to 1, while the HR for SICP and TLEI in the delayed phase exceeds 1. Based on univariate analysis, none of these parameters reached statistical significance. Among 39 deaths (40.2% of the cohort), 53.8% (*n* = 21) occurred in patients with high SICP (≥ 90.3%), while 46.2% (*n* = 18) had low SICP. 68 (70.10%) of the 97 patients experienced tumor recurrence (local or metastatic).

**Table 5:** Multivariable-adjusted hazard ratio (HR) for high SICP values in CRLM after multiple chemotherapy regimens

Dependent:		All	HR (univariable)	HR (multivariable)
PFF	0	17 (17.5)	-	-
	1	80 (82.5)	0.55 (0.28–1.07, p = 0.078)	0.43 (0.21–0.89, p = 0.023)
AGE	Mean (SD)	62.7 (10.6)	0.98 (0.96–1.01, p = 0.219)	0.97 (0.94–1.00, p = 0.023)
SICP (delayed)	Mean (SD)	46.6 (36.8)	1.01 (1.00–1.01, p = 0.090)	1.01 (1.00–1.01, p = 0.043)
TLEI (delayed)	Mean (SD)	1.1 (0.9)	1.29 (0.99–1.70, p = 0.063)	1.38 (1.05–1.82, p = 0.023)
Dependent:		All	HR (univariable)	HR (multivariable)
AFF	0	17 (17.5)	-	-
	1	80 (82.5)	0.55 (0.28–1.07, p = 0.078)	0.45 (0.22–0.92, p = 0.029)
AGE	Mean (SD)	62.7 (10.6)	0.98 (0.96–1.01, p = 0.219)	0.97 (0.94–0.99, p = 0.019)
SICP (delayed)	Mean (SD)	46.6 (36.8)	1.01 (1.00–1.01, p = 0.090)	1.01 (1.00–1.01, p = 0.043)
TLEI (delayed)	Mean (SD)	1.1 (0.9)	1.29 (0.99–1.70, p = 0.063)	1.38 (1.05–1.82, p = 0.023)

Chemotherapy groups: PFF—panitumumab + FOLOX-4 and/or FOLFIRI

AFF—aflibercept + FOLFOX-4 and/or FOLFIRI

## Discussion

This study presents the quantitative MRI-based protocol for evaluating fibrosis in treated CRLM. By employing hepatobiliary-phase Gd-BOPTA-enhanced imaging we were able to identify and quantify delayed enhancement in CRLM, presumably related to fibrosis-associated changes with increased collagen deposition in CRLM. For this purpose, we utilized the SICP at 5-min and 60 min – the last parameter emerged as a particularly reliable marker.

The rationale for quantitatively assessing fibrosis in CRLM using MRI arises from the urgent need for objective, reproducible, and clinically relevant biomarkers in oncologic imaging. Conventional fibrosis evaluation in CRLM was restricted to histopathology, which, despite being the gold standard, is invasive, susceptible to sampling bias, and impractical for longitudinal monitoring [26]. Furthermore, previous imaging studies focusing on the assessment of fibrosis in CRLM primarily depended on qualitative or semi-quantitative evaluations, which can be affected by interobserver variability and limited reproducibility [4, 8]. Our SICP/TLEI approach complements existing non-invasive fibrosis biomarkers but differs in key aspects. While elastography (MR elastography) quantifies tissue stiffness as a surrogate for fibrosis, it cannot discriminate tumor-specific stromal changes from background liver fibrosis. Diffusion-weighted imaging (DWI) captures cellularity but lacks specificity for collagen deposition. In contrast, SICP leverages gadobenate dimeglumine's extracellular retention in fibrotic networks, potentially offering direct insight into tumor-associated fibrosis.

Recent evidence provides a biologically plausible mechanism linking SICP/TLEI-derived fibrosis to poor outcomes in CRLM. High SICP/TLEI values likely reflect a desmoplastic, collagen-rich stroma that limits drug diffusion and oxygen penetration, creating hypoxic microdomains that stabilize HIF-1α and activate downstream pathways promoting angiogenesis, metabolic adaptation, and epithelial–mesenchymal transition [27–29]. In this setting, cancer-associated fibroblasts (CAFs) become persistently activated through TGF-β/HIF-1α signaling, producing additional extracellular matrix components and immunosuppressive mediators (e.g., CXCL12, IL-6), which exclude cytotoxic lymphocytes and dampen anti-tumor immunity [30, 31]. This fibrosis–hypoxia–immunosuppression loop fosters chemoresistance and recurrence, consistent with preclinical models of pancreatic and hepatic carcinomas where dense stroma predicts limited therapeutic response [32, 33]. In our cohort, patients receiving anti-EGFR therapy showed lower SICP/TLEI values and improved survival compared with those treated with anti-angiogenic regimens, suggesting therapy-specific modulation of stromal remodeling.

While tumor fibrosis was historically viewed as a morphological indicator of therapeutic efficacy, our findings suggest a paradoxical relationship between post-chemotherapy fibrosis and worse overall survival [34–36]. This observation aligns with recent histological evidence from Ikuta et al., which indicated that increased fibrosis, evidenced by collagen deposition and α-smooth muscle actin (α-SMA) expression in metastatic lymph nodes (MLNs), independently predicts reduced survival in colorectal cancer patients. Notably, α-SMA-positive stromal cells in MLNs co-expressed podoplanin, confirming their identity as fibroblastic reticular cells (FRCs) – mesenchymal elements recognized for their role in shaping immunosuppressive environments [29]. Our findings align with emerging evidence across malignancies where stromal biomarkers predict outcomes: in breast cancer, MRI-derived textural features and diffusion restriction in fibrotic foci correlate with triple-negative subtype, chemoresistance, and early recurrence; in gastric cancers with elevated elastography scores (indicative of stromal rigidity) demonstrate higher peritoneal metastasis rates, analogous to the metastatic progression observed here in high-SICP/TLEI subgroups. Notably, SICP/TLEI differs from purely structural biomarkers (CT density) by probing microstructural integrity through water diffusion constraints in fibrotic networks. This positions it closer to functional biomarkers like DWI/ADC yet with distinct contrast mechanisms sensitive to collagen architecture.

The choice to quantitatively assess fibrosis in CRLM using MRI stems from the critical need for objective, reproducible, and clinically relevant biomarkers in oncologic imaging. MRI is frequently employed for characterizing indeterminate liver lesions, and its function in staging liver fibrosis has gained increasing recognition. Compared to computed tomography (CT), MRI offers superior soft tissue contrast without involving ionizing radiation; however, it is associated with longer acquisition times and susceptibility to respiratory motion artifacts [37, 38].

Previous research suggests that collagen deposition could be present in hepatic metastases and chemotherapy-induced fibrosis may obscure viable tumor remnants, promote hypoxic conditions, and enhance tumor resistance [32]. Delayed contrast enhancement on MRI likely reflects these fibrotic changes, thereby serving as a non-invasive biomarker for aggressive disease biology. Although the SICP ≥ 90.3% threshold was initially adopted as a hypothesis-generating reference, the present analysis provides internal validation supporting its applicability in CRLM. ROC-based cutoff optimization within the study cohort identified an optimal threshold of approximately 90%, and survival associations remained stable across a narrow cutoff range. These findings indicate that the prognostic value of SICP is not driven by arbitrary dichotomization but reflects a biologically meaningful continuum of delayed contrast retention associated with tumor fibrosis [27–29]. While the biological differences between renal tumors and colorectal liver metastases are acknowledged, the current findings suggest that delayed gadolinium retention reflects shared extracellular matrix properties rather than organ-specific perfusion alone [9]. The internally validated cutoff supports the relevance of SICP as a liver-specific imaging biomarker, despite its conceptual origins in renal oncology literature [9]. In analogy to previous studies on renal lesions, where a cutoff value of SICP ≥ 90.3% in the excretory phase was associated with significant fibrosis, our results suggest that similarly elevated SICP and TLEI values in liver metastases – particularly among patients after chemotherapy– may reflect therapy-induced fibrotic remodeling [9]. Although internal ROC analysis supports a threshold near 90%, this value should be interpreted as hypothesis-generating. Validation in prospective multicenter cohorts is warranted to confirm its prognostic relevance in CRLM.

Incorporating quantitative fibrosis assessment into routine imaging may be challenging, however could provide a reproducible and clinically valuable metric for treatment response [39]. Unlike histopathology, which remains invasive and impractical for serial assessment, MRI offers a framework for longitudinal monitoring of fibrosis. Nonetheless, standard MRI protocols may struggle to differentiate between fibrosis and necrosis or desmoplastic reactions, necessitating further optimization of imaging sequences [40].

This study has several limitations that should be acknowledged. First, its retrospective design and inclusion of a chemotherapy-treated population may have introduced an overrepresentation of tumors exhibiting pronounced stromal remodeling. A major limitation of this study is the restricted number of patients for whom radiologic–pathologic correlation was feasible. This constraint was structural and not remediable: only lesions that were surgically resected in precise anatomical correspondence to the preoperative MRI could be included, and in many patients, multifocal resections, ablation procedures, interval tumor evolution, or merging of lesions prevented accurate one-to-one matching. In addition, no further archival specimens from the study period were available for retrospective evaluation. As a result, expanding the pathology cohort was not possible, and this inherently limited the depth of histopathologic validation. Nevertheless, the available cases demonstrated a consistent stepwise relationship between fibrosis grade and SICP/TLEI, supporting the internal validity of our findings. Although the correlation between SICP and histologic fibrosis is biologically plausible, the extent to which these findings can be generalized remains unclear. The cohort’s treatment heterogeneity – particularly with regard to varying chemotherapy regimens (FOLFOX-4 and others) – limited the ability to perform regimen-specific analyses. Consequently, the observed associations are primarily reflective of outcomes within the FOLFOX-4 subgroup, a regimen known to induce fibrotic changes, potentially skewing the findings toward more aggressive or treatment-resistant tumors. Tumor heterogeneity – including variations in perfusion, necrosis, or cellularity – may also influence SICP values independently of fibrosis, potentially limiting the specificity of this imaging biomarker. Furthermore, the single-center nature of the study, combined with a moderate sample size and the lack of functional imaging data, may restrict the external validity of the results. Technical limitations also warrant consideration. SICP measurements are susceptible to respiratory motion artifacts, particularly in lesions located near the diaphragm. Despite standardized breath-hold protocols, motion-related degradation led to the exclusion of 15 otherwise eligible scans, which may have introduced an additional layer of selection bias. Future studies should incorporate advanced motion-compensated imaging techniques, such as free-breathing sequences with compressed sensing, to enhance measurement reliability. An important methodological constraint lies in the use of three different MRI platforms (1.5 T and 3 T systems from multiple vendors), without prior inter-scanner validation. Variability introduced by differing field strengths and manufacturer-specific parameters challenges the comparability of SICP values across scanners and limits the feasibility of pooled analyses. Prospective studies should include cross-platform reproducibility assessments in a representative patient subgroup to ensure measurement consistency and should implement semi-automated VOI-based quantification to improve accuracy and representation of tumor heterogeneity. Although MRI was consistently performed 4–6 weeks after chemotherapy completion, the temporal evolution of fibrosis remains dynamic, and imaging at earlier or later intervals might yield divergent SICP values. Longitudinal imaging with serial MRI assessments could provide a more comprehensive understanding of fibrosis kinetics and inform optimal timing for evaluation. Lastly, while the reproducibility of SICP–TLEI associations across univariate and multivariate models supports their biological relevance, the unadjusted p-values should be interpreted as exploratory, and not definitive, given the absence of correction for multiple testing. Importantly, the consistency of results across OS and RFS endpoints, multivariate models, and cutoff sensitivity analyses reinforces the robustness of SICP as a prognostic imaging biomarker in CRLM.

## Conclusion

The degree of contrast enhancement of CRLM during hepatobiliary phase after Gd-BOPTA injection, following chemotherapy may correlate with tumor fibrosis, as indicated by increased SICP and TLEI values and could be regarded as potential imaging biomarkers for evaluating treatment response and prognosis. However, given that our results indicate a negative association between high SICP values and overall survival – contrary to previous studies – additional large-scale, prospective research is essential to clarify the clinical significance of fibrosis in CRLM.

## Supplementary Information

Below is the link to the electronic supplementary material.

Supplementary file1 (DOCX 34 KB)

## Data Availability

The datasets generated and/or analysed during the current study are not publicly available due to the presence of sensitive personal data but are available from the corresponding author upon reasonable request.
